# Predictive Models of Morbidity and Mortality in Gynaecological Cancer Surgery: A Systematic Review

**DOI:** 10.3390/jcm15207948

**Published:** 2026-10-14

**Authors:** Katie Avelina Fernandes, Sonu Krishel Vidanagamage, Mevan Gooneratne, Robert Wilkinson, Madeleine Wells, Ching Ling Pang, Shaun May, Georgia Alexandra Elizabeth Ellis, Elly Brockbank, Arjun Jeyarajah, Alexandra Lawrence, Saurabh Phadnis, Ranjit Manchanda, Samuel George Oxley, Michail Sideris

**Affiliations:** 1 Barts and The London School of Medicine and Dentistry, Queen Mary University of London, London E1 2AD, UK; k.fernandes@smd22.qmul.ac.uk (K.A.F.);; 2 Department of Anaesthesia, Pain and Peri-Operative Medicine, Royal London Hospital, Barts Health NHS Trust, London E1 1FR, UK; 3 Department of Gynaecological Oncology, Royal London Hospital, Barts Health NHS Trust, London E1 1FR, UKr.manchanda@qmul.ac.uk (R.M.);; 4 Wolfson Institute of Population Health, Queen Mary University of London, Charterhouse Square, London EC1M 6BQ, UK

**Keywords:** gynaecological oncology, clinical prediction model, risk stratification, postoperative complications, mortality, cytoreductive surgery, frailty, external validation

## Abstract

**Background/Objectives**: Gynaecological–oncological surgery involves substantial perioperative morbidity and mortality risks. Reliable preoperative risk stratification could inform consent, shared decision-making and perioperative optimisation. Several prediction models exist, but their performance and methodological quality in this population remain uncertain. We aimed to provide a comprehensive synthesis of existing evidence to guide the selection and development of prediction tools in this population. **Methods**: We searched MEDLINE and EMBASE using a prospective protocol (PROSPERO CRD420251179495) and included studies developing or validating multivariable prediction models or risk scores for graded postoperative morbidity/mortality after gynaecological–oncological surgery. Risk of bias and applicability were assessed using the Prediction model Risk Of Bias Assessment Tool (PROBAST) at the study (publication) level. Findings were synthesised narratively. **Results**: Twenty-eight studies (75,999 patient records, including overlapping cohorts) were included (23 retrospective, five prospective; sample sizes 100–21,359). Models were multifactorial (*n* = 17), including generic and gynaecological–oncological-specific tools, frailty-based (*n* = 8) models or biomarker and index-based (*n* = 3) models. A numerical c-statistic was reported in 20 studies (binary c-statistics 0.50–0.92 across heterogeneous outcome definitions), calibration was reported in nine studies, and internal validation was reported in thirteen. No frailty-based model reported calibration or validation. One model specific to ovarian cytoreductive surgery was independently externally validated, retaining discrimination (c-index 0.715) with reduced calibration. Generic tools performed poorly, (c-statistics ranged from 0.529-0.644). Twenty studies were at high risk of bias, predominantly within the analysis domain. **Conclusions**: Existing models show, at best, moderate discrimination, limited calibration and infrequent external validation within the gynaecological–oncological surgical population. No model can currently be recommended for routine clinical practice. Further independent external validation of specialty-specific models in local settings, with full calibration reporting, is needed to support preoperative counselling.

## 1. Introduction

Gynaecological cancers, including cervical, endometrial, ovarian, vulval and vaginal, accounted for an estimated 1.47 million new diagnoses and 680,372 deaths worldwide in 2022 [1]. Surgical resection is central to management, yet major gynaecological–oncological (GO) surgery can involve high perioperative risk [2,3]. Reliable preoperative risk stratification can inform consent and shared decision making and guide preoperative optimisation and perioperative care.

Multivariable prediction models estimate individual risk from routinely available clinical variables. Surgical risk models have evolved from procedure-specific tools towards procedure-independent instruments, e.g., the Physiological and Operative Severity Score for the enUmeration of Mortality and Morbidity (POSSUM) [4] and its Portsmouth modification (P-POSSUM) [5] and, subsequently, registry-derived calculators such as the American College of Surgeons National Surgical Quality Improvement Program (ACS-NSQIP) surgical risk calculator [6]. Because these tools were derived from heterogeneous surgical populations, their applicability to GO surgery is uncertain [7]. GO surgery poses a number of unique challenges, as procedures are complex and frequently undertaken in older, comorbid and frail patients [8,9], whose disease extent varies widely. As a result, generic tools have shown variable and often reduced discrimination in GO cohorts and specialty-specific risk-assessment tools have been advocated [10].

Clinical utility depends on the ability to rank patients by risk (discrimination), measured using the area under the receiver operating characteristic curve (AUROC or c-statistic), the agreement between predicted and observed risk (calibration), and adequate validation to limit optimism and overfitting [11]. A model may discriminate well yet be poorly calibrated, systematically over- or underestimating risk, which would limit its use in clinical practice.

Numerous specialty-specific and augmented models have since been developed, including frailty indices, but methodological quality and reporting vary [12,13]. This uncertainty may contribute to the lack of uptake amongst gynae–oncologists, as well as the desire for specialty-specific tools [14].

Previous reviews have mostly examined individual prognostic factors rather than multivariable prediction models [15], and frailty has not been consistently evaluated within structured prediction models. To date, no systematic review has comprehensively evaluated the development, validation and performance of prediction models for postoperative morbidity and mortality in GO surgery. This study therefore aimed to systematically identify and critically appraise studies developing or validating models predicting 30-day morbidity and/or mortality after gynaecological–oncological surgery, as well as to appraise their risk of bias and applicability.

## 2. Materials and Methods

### 2.1. Protocol and Registration

The review followed a prospective protocol (PROSPERO CRD420251179495) and is reported according to the PRISMA 2020 statement (Supplementary S.1) [16].

### 2.2. Eligibility Criteria

**Figure 1:**
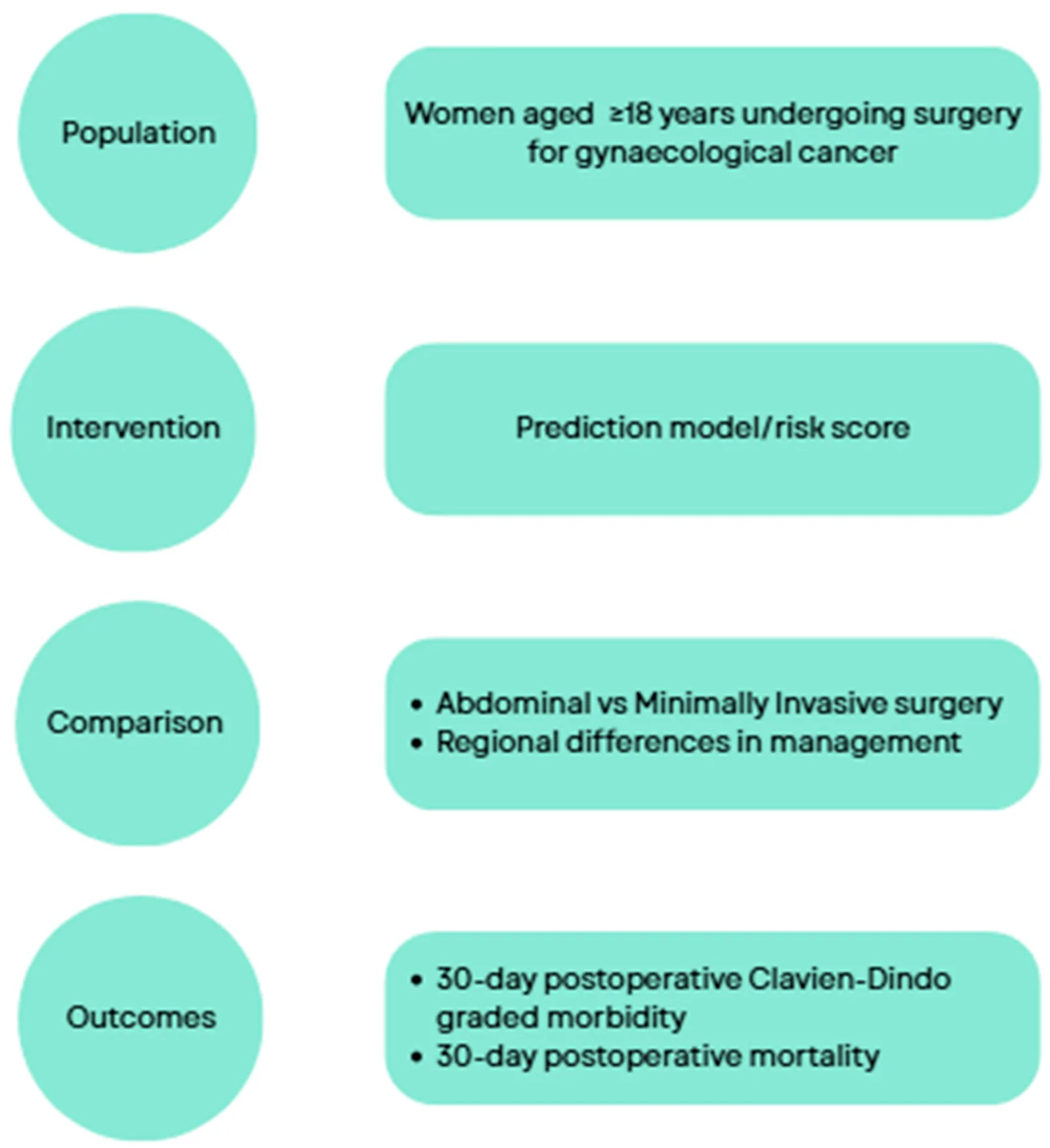
Population, intervention, comparison and outcomes (PICO) framework used to define study eligibility. The corresponding PICOTS elements are described in Section 2.2.

Eligibility was defined using a population, intervention, comparison, and outcomes (PICO) framework (Figure 1). Consistent with guidance for reviews of prediction-model studies [13], these criteria correspond to the PICOTS elements: population, adults undergoing GO surgery; index model(s), any multivariable prediction model or risk score; comparator(s), none prespecified, although existing generic tools were eligible where evaluated in GO cohorts; outcome(s), graded postoperative morbidity and/or mortality; timing, prediction intended before surgery for outcomes within 30 days of surgery; and setting, hospital-based GO surgical care. Models incorporating intraoperative or postoperative predictors were eligible but are identified as perioperative rather than preoperative tools (Section 3.3). We included studies of women aged ≥ 18 years undergoing surgery for confirmed or suspected ovarian, cervical, endometrial, vaginal or vulval cancer that developed and/or validated a multivariable prediction model or risk score for graded 30-day postoperative morbidity and/or mortality, with complication severity classified using the Clavien–Dindo system or a recognised derivative (e.g., Accordion; Memorial Sloan Kettering Cancer Center [MSKCC] system). Under the Clavien–Dindo classification, grades I–II denote complications managed pharmacologically or with minor intervention; grades III–IV are major deviations requiring surgical, endoscopic or radiological intervention or organ support; and grade V is death [17]. A prediction model or risk score was defined as a combination of two or more predictors, including composite indices such as frailty scores, used to estimate or stratify an individual’s risk of the outcome. All study designs were eligible. The 30-day window was chosen as the conventional reporting period for postoperative morbidity and mortality in surgical outcome registries and risk calculators [6]. Where a study reported a longer maximum follow-up period, only outcomes occurring within 30 days of surgery were extracted.

We excluded studies that evaluated prognostic factors without a multivariable model or risk score, assessed a single complication only, did not grade complication severity, did not report 30-day outcomes, combined GO patients with non-GO patients (e.g., benign gynaecology, colorectal surgery) in a manner precluding separate analysis, included only the abstract, or that were review articles.

### 2.3. Literature Search

We searched MEDLINE and EMBASE on 20 November 2025 using a predefined strategy combining terms for gynaecological cancer, surgery, postoperative morbidity and mortality, as well as prediction models or risk scores, in English, with no date restriction (Supplementary S.2). Search sensitivity was confirmed against eligible studies identified a priori by senior authors (S.G.O., M.S.) [18,19].

### 2.4. Study Selection

Records were imported into EndNote v20.2 (Clarivate, Philadelphia, PA, USA) and de-duplicated. Two reviewers (K.A.F., S.K.V.) independently screened titles and abstracts, then full texts, against the eligibility criteria. Disagreements were resolved by discussion and, where necessary, adjudication by senior authors (S.G.O., M.S.).

### 2.5. Data Extraction

Two reviewers (K.A.F., S.K.V.) extracted data independently using a piloted standardised form. Extracted items were informed by the Transparent Reporting of a multivariable prediction model for Individual Prognosis or Diagnosis (TRIPOD) statement [12]; these included study design, country, and data source; population and eligibility criteria; surgical approach and procedure; model type, candidate and final predictors, and modelling method; outcome definition and grading system; and measures of discrimination, calibration, and validation. Where a study reported more than one model, each was extracted separately. Because only aggregate data were available, patients contributing to more than one study (e.g., overlapping ACS-NSQIP cohorts) could not be identified or de-duplicated at the individual level; overlapping cohorts were therefore identified by data source and study period, each study was extracted as reported, and patient totals were presented as a descriptive sum of records rather than of unique patients. As no data were pooled, this overlap does not affect any quantitative estimate. Outcome events per predictor (EPP) were calculated as the number of outcome events divided by the number of predictors reported for each model.

### 2.6. Quality Assessment

Two reviewers (K.A.F., S.K.V.) independently assessed the included studies using the Prediction model Risk of Bias Assessment Tool (PROBAST) [13], across the participants, predictors, outcomes, and analysis domains, rating both risk of bias and applicability; disagreements were resolved by discussion with senior authors (M.S., S.G.O.). PROBAST was applied at the study (publication) level; where a study reported more than one model or analysis, a single judgement was made for each domain across the reported analyses. Assessments were made manually against the published PROBAST signalling questions and recorded on a standardised form; no software was used for the risk-of-bias assessment or the narrative synthesis as no statistical pooling was undertaken.

### 2.7. Synthesis

Substantial clinical and methodological heterogeneity was anticipated, and meta-analysis was not planned. We undertook a structured narrative synthesis, tabulating study characteristics, populations, and procedures, as well as model characteristics and performance, according to the PICO framework. Discrimination was summarised separately by target outcome (mortality, severe morbidity and broader morbidity endpoints), and multiclass measures were not compared with binary c-statistics.

## 3. Results

### 3.1. Study Characteristics

**Figure 2:**
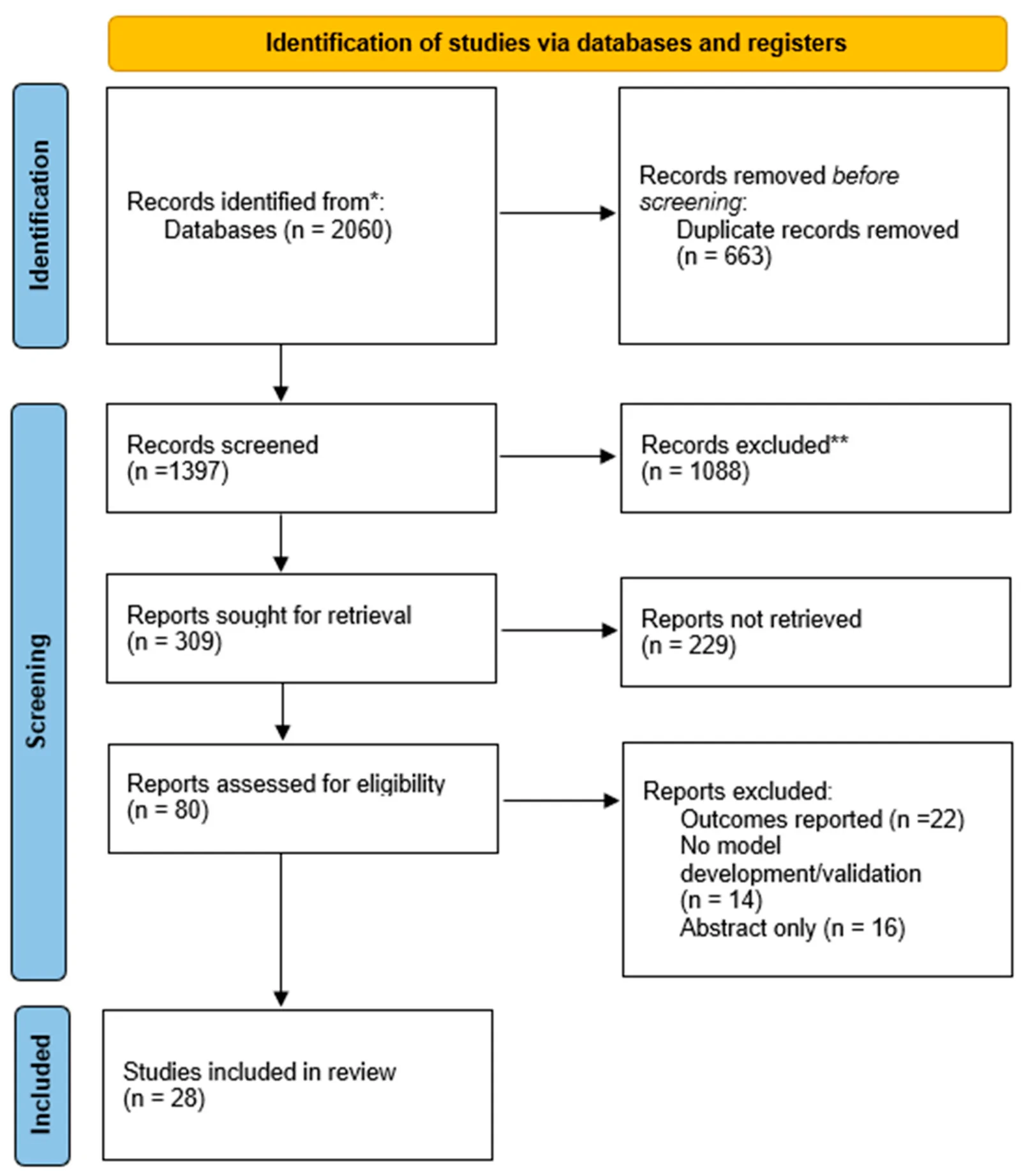
PRISMA 2020 flow diagram of study selection [16]. * Records identified from database searching. ** Records excluded on title and abstract screening.

The search returned 2060 records; after the removal of 663 duplicates, 1397 titles and abstracts were screened, and 80 full-text reports were assessed, of which 28 studies met the eligibility criteria, including 75,999 patients (with overlapping cohorts) (Figure 2; Table 1) [10,18,19,20,21,22,23,24,25,26,27,28,29,30,31,32,33,34,35,36,37,38,39,40,41,42,43,44]. The studies were conducted in Europe, Asia and North America, with patient records from 2003 to 2023. Twenty-three used a retrospective and five a prospective cohort design [18,27,34,36,42]. Sample sizes ranged from 100 [25] to 21,359 [38]. Six studies from the USA used the ACS-NSQIP registry [21,22,26,29,32,33]; for Hermann, only the GO subgroup (*n* = 21,215) was eligible [26]. Three studies reported a follow-up duration exceeding 30 days [20,25,28]. Alci reported follow-up (mean, median, SD) within 30 days postoperatively (longest follow-up: 77 days; outliers not specified) [20]. Jiang reported 30-day morbidity and 90-day mortality [28]. Giannini did not report a formally defined 30-day endpoint but reported that 80% of postoperative complications had been resolved within 30 days [25]; this study was therefore treated as a borderline eligibility case, and its inclusion by reviewer consensus at full-text screening represents a deviation from the prespecified protocol. In a sensitivity check excluding this study, the review would comprise 27 studies (75,899 patient records), including seven frailty-based studies and 19 studies at high risk of bias; as it reported no discrimination, calibration or validation measures, no performance estimate or conclusion of this review would change.

### 3.2. Population

Twenty-three studies (82%) included patients with ovarian cancer (Table 2). Where reported, the International Federation of Gynaecology and Obstetrics (FIGO) stage was grouped as early (I–II) or advanced (III–IV); mixed gynaecological-cancer cohorts contained a higher proportion of early-stage disease than ovarian-specific cohorts, which were predominantly advanced-stage. Eleven studies did not report FIGO stage [10,19,21,22,23,26,27,29,32,33,40]. Mean or median age ranged from 43 to 75 years, most commonly in the sixth decade; ages were approximated where studies reported grouped age categories. Most patients underwent open surgery, most frequently cytoreductive surgery (primary or interval).

### 3.3. Model Characteristics

Twenty-four studies used multivariable regression, predominantly logistic (two used Cox regression [19,39]); one used linear-by-linear association [40]; two used machine-learning methods (Refs. [18,20], the latter alongside logistic regression); and one applied an existing calculator without developing a new model, reporting performance using the Brier score [43] (Table 3). Models were classified as multifactorial (*n* = 17) [10,18,19,20,21,22,24,28,29,31,32,39,40,41,42,43,44], frailty-based (*n* = 8) [23,25,26,27,33,34,37,38], or biomarker and index-based models (*n* = 3) [30,35,36]. Fifteen studies developed a new model and thirteen validated or applied existing tools; multifactorial models were the most common type, whereas frailty-based models were more often validation studies. Five frailty studies used the 11-item modified frailty index (mFI) or its five-item derivative [23,25,26,33,37] (Table S1).

Four studies evaluated existing generic tools: the P-POSSUM [41], the ACS-NSQIP surgical risk calculator [10,43] and the modified surgical Apgar score [40]. Four studies undertook external validation in GO cohorts: three evaluated generic surgical tools (the ACS-NSQIP surgical risk calculator [10,43] and P-POSSUM [41]), and one independently validated a GO-specific model, the nomogram of Cham and colleagues [22], in a separate institutional cohort [42].

The number of predictors ranged from two to forty-nine; nine models included more than ten predictors [10,18,20,23,25,29,34,38,43]. The most frequent predictors were age, comorbidity, surgical complexity, body mass index, functional status and cancer stage. Seven models incorporated intraoperative or postoperative variables, such as operative time, estimated blood loss, intraoperative haemodynamics or length of stay [10,18,19,20,29,40,43], and were therefore perioperative rather than strictly preoperative tools. Complication severity was graded with the Clavien–Dindo classification in most studies, the Accordion classification in two [28,31], and the MSKCC system in one [44]; most models defined major morbidity as having a Clavien–Dindo grade ≥ III (Table 3).

Fourteen models of 30 (47%) had fewer than 10 outcome events per predictor (EPP) [18,20,23,24,25,27,30,34,35,37,40,41,42,43] (Table 3). Although no single EPP threshold guarantees an adequate sample size, this indicates limited outcome events relative to model complexity and a potential risk of overfitting [13].

The most frequently reported outcomes were major morbidity and 30-day mortality. The frequency of Clavien–Dindo grade III and above events has been included in Table S2.

### 3.4. Overview of Model Performance

A numerical measure of discrimination (AUROC/c-statistic) was reported in twenty studies (Table 4). The highest morbidity estimate (0.94) is a multiclass AUROC from the neural network in the GO-specific GO-SOAR calculator; the corresponding logistic-regression model achieved 0.66 for mortality [18]. The lowest value (0.5) came from an ACS-NSQIP universal surgical risk calculator [43]. Eight studies did not report a numerical c-statistic [23,24,25,27,33,34,36,40].

Because predicted outcomes ranged from any complication to mortality alone (Table 3), c-statistics are not directly comparable across models and are therefore summarised by target outcome. For 30-day mortality, binary c-statistics ranged from 0.66 to 0.86 [10,18,26,29]; for severe morbidity (Clavien–Dindo ≥ III or equivalent, including composite severe morbidity/mortality endpoints), from 0.50 to 0.92 [10,20,21,22,26,28,29,30,31,32,37,42,43,44]; and for broader morbidity endpoints (Clavien–Dindo ≥ I or ≥II), from 0.529 to 0.723 [10,19,35,38,39,41]. The multiclass AUROC of 0.94 for GO-SOAR [18] is not comparable with these binary estimates.

Internal validation was assessed in thirteen studies, most often by bootstrapping [18,19,20,22,28,30,31,32,35,39,41,42,44]; none of the eight frailty studies did so. Calibration was reported in nine studies [19,21,22,28,31,35,41,42,44]. Of these, five presented a calibration plot or curve of predicted versus observed risk [22,28,31,41,42], whereas four reported only a Hosmer–Lemeshow test [19,21,35,44], which provides limited information on the magnitude and direction of miscalibration [11]. No frailty study reported calibration, and among biomarker and index-based models, only one did [35].

### 3.5. Multifactorial Models (n = 17)

Multifactorial models fell into three main groups: generic surgical models applied to the GO cohort, ovarian cytoreductive surgery-specific models, and GO surgical models. Two generic surgical models were assessed for validation in GO. The ACS-NSQIP calculator showed poor discrimination, with a c-statistic of 0.5–0.644 for major morbidity [10,43], although a higher value of 0.851 for mortality (however, based only on nine recorded deaths) [10]. P-POSSUM was also found to have low discrimination in cytoreductive surgery for ovarian cancer (c = 0.529), with poor calibration; this was improved to 0.655 by the addition of the Edmonton Frail Scale and body mass index [41].

Several models were developed specifically for ovarian cytoreductive surgery. Cham and colleagues’ nomogram for predicting Clavien–Dindo IV–V complications achieved an internal c-index of 0.71, with bootstrap internal validation, and good internal calibration [22]; this underwent independent external validation with almost identical discrimination (c-index 0.715), although with slightly reduced calibration [42]. Other models showed moderate discrimination, including from Kumar (0.648) [31], Latif (0.66) [32], Nieuwenhuyzen-de Boer (0.62) [39], Jiang (0.75 overall, 0.79 for severe complications) [28], and Vizzielli (0.79), who each used bootstrapping or tenfold cross-validation for internal validation, as well as Barber (0.725) who did so without internal validation; all provided no external validation. The machine-learning model of Alci demonstrated an AUROC of 0.863 for Clavien–Dindo grade ≥ III complications, with internal validation on a random split sample, although it did not report calibration or external validation; this incorporated intraoperative and postoperative variables and so is less applicable to preoperative risk prediction [20].

Among more general GO models, an NSQIP-based model for hysterectomy reported a c-statistic of 0.86 for mortality and 0.68 for major morbidity, without reporting internal validation, calibration, or external validation [29]. Shim and colleagues reported a model with a discrimination of 0.656, internal validation of 0.674, and calibration (non-significant Hosmer–Lemeshow test) [19]. The GO-SOAR (PROMEGO) calculator is reported to be the only prospectively developed multicentre model [18]; its neural-network variant achieved a multiclass AUROC of 0.94 for morbidity, whereas the interpretable logistic-regression model (the form intended for clinical use) achieved a more modest 0.66 for mortality. Neither calibration nor external validation were reported. The modified surgical Apgar score for robotically assisted radical hysterectomy for cervical cancer did not report discrimination, calibration, internal or external validation [40]. This study was judged as having a high risk of bias. Other than the nomogram by Cham and colleagues, no GO-specific model reported external validation.

### 3.6. Frailty-Based Models (n = 8)

Eight studies evaluated frailty-based models; two using the mFI-11 [23,25], three using the mFI-5 [26,33,37], one using the Fried Frailty Score [27], one using the Tilburg Frailty Indicator [34], and one using the preoperative frailty index and the Johns Hopkins Adjusted Clinical Groups frailty indicator [38]. The mFI-5 showed moderate discrimination for Clavien–Dindo III–IV complications (c = 0.732) and mortality (c = 0.801) in one study [26], and in another, it achieved an adjusted c-statistic of 0.92 for Clavien–Dindo III–V complications in a multivariable model including age and body mass index [37]; this estimate reflects the combined multivariable model rather than the frailty index alone. Moderate discrimination was seen for the preoperative frailty index (0.71) and the Adjusted Clinical Groups frailty indicator (0.70) [38]. Others did not report discrimination, and none reported calibration, internal or external validation.

### 3.7. Biomarker and Index-Based Models (n = 3)

Three models were based on a single dominant index or biomarker, all in ovarian cancer. The Peritoneal Cancer Index was assessed for discrimination graphically, but no numerical c-statistic was reported [36]. Köse and colleagues evaluated several preoperative inflammatory factors; at best, these showed moderate discrimination for CA125 (0.766). The Systemic Immune–Inflammation Index showed a c-statistic of 0.740 and used bootstrapping for internal validation but did not report calibration [30]. None were externally validated.

### 3.8. Surgical Setting

Most models were developed for ovarian cytoreductive surgery, and those studies that reported external validation did so predominantly in this cohort [10,41,42,43]. Two studies focused on vulval cancer, neither of which reported discrimination [24,33]. One study focused exclusively on robotic-assisted radical hysterectomy for cervical cancer, which used intraoperative predictors [40]. Other studies included mixed GO cohorts, including most GO cancer types.

### 3.9. Risk of Bias

Using PROBAST, applied at the study level (Table S3), twenty studies were judged as having a high risk of bias, primarily within the analysis domain, due to an absence of model validation or calibration, incomplete reporting of discrimination, confidence intervals, and missing data or few outcome events per predictor [10,18,20,21,23,24,25,26,27,29,30,32,33,34,36,37,38,39,40,43].

Additionally, seven of these had a potential risk of bias in the participant or predictor domains [10,20,21,34,37,38,40]. Studies that restricted adults to ages over 65 [21] or 70 [37] were at risk of skewed morbidity and mortality outcomes relative to studies with broader age ranges. Given that the GO population is commonly co-morbid, one study with excessive exclusion of patients due to underlying disease (e.g., taking antidepressants, cognitive dysfunction, hearing impairments, neurological disease) raised concern [34]. Three studies reporting models with 20 or more predictors [10,20,38] were at risk of overfitting and reduced clinical feasibility, given the limited number of outcome events for model development. In one study, the inclusion of intraoperative predictors also increased the risk of bias, as models are intended for risk stratification and preoperative use [40].

Eight studies were judged as having a low risk of bias across all four domains [19,22,28,31,35,41,42,44]. No study raised outcome domain or applicability concerns, as models fit the review definition (Table S3).

## 4. Discussion

### 4.1. Main Findings

This systematic review identified 28 studies developing or validating multivariable models for predicting 30-day postoperative morbidity or mortality after GO surgery, including multifactorial, frailty-based, biomarker-based, and index-based models. The generic surgical-risk calculators ACS-NSQIP and P-POSSUM performed poorly in the GO surgical population. Frailty-based scores showed at most moderate discrimination for adverse outcomes (c = 0.70–0.80), with a higher estimate reported in a single study when combined with other patient factors, but no frailty study reported calibration or validation. Several GO-specific risk calculators have been developed, some of which show moderate discrimination. One model for ovarian cytoreductive surgery has been externally validated; another prospectively developed international model for GO surgery has not yet reported calibration or external validation in the included studies. No model currently demonstrates adequate discrimination, calibration, and external validation and can be recommended for all GO patients.

### 4.2. Strengths and Limitations

This is the first systematic review to evaluate the performance of major morbidity and mortality prediction models within the GO surgical population. It followed a prospectively registered protocol and PRISMA 2020 [16], with duplicate screening, extraction and PROBAST appraisal. Its reporting was also checked against TRIPOD-SRMA, the reporting guideline for systematic reviews of prediction-model studies [45]. As expected, there was substantial clinical and methodological heterogeneity in populations, procedures, predictors, and outcome definitions that precluded meta-analysis, and therefore, we performed a narrative synthesis. Two bibliographic databases were searched and were restricted to English-language reports without citation tracking, grey-literature searching, or contact with study authors; therefore, a small number of eligible studies may have been missed. The overall population is a descriptive sum rather than a count of distinct patients: six included cohorts drew on the ACS-NSQIP registry over overlapping periods, so individuals are probably counted more than once, and the figure should not be interpreted as independent participants. The reporting of calibration, discrimination and other factors was inconsistent, and risk of bias could not be fully assessed in every domain. PROBAST was applied at the study level rather than the model or analysis level, so differences in risk of bias between models or between development and validation analyses within the same study may not be fully captured. The inclusion of one study without a formally defined 30-day endpoint [25] was a deviation from the protocol, although a sensitivity check excluding it did not alter any result (Section 3.1). Several frailty- and biomarker-based studies evaluated a composite index rather than developing a model intended to generate individual risk estimates; these were retained because such indices are themselves multivariable scores used for risk stratification, but they lie at the boundary between prediction-model and prognostic-factor research and should be interpreted accordingly. Finally, EPP was calculated from the number of predictors reported because candidate predictors and predictor parameters were inconsistently described and may therefore overstate the effective sample size available for model development.

### 4.3. Interpretation

The methodological quality of included studies limits conclusions. Many models were developed in small single-centre cohorts with few outcome events, giving few outcome events per predictor, which increases the risk of overfitting, e.g., the c-statistic of 0.851 for mortality in nine events [10]. This highlights the importance of external validation, which was only undertaken in one GO-specific model. Internal validation was often absent, and calibration was not always reported. Several models included intra/postoperative variables, which precludes their use preoperatively. These limitations are the main barriers to widespread implementation.

It is important for clinicians working in GO services to be aware of the distinction between discrimination and calibration. A high c-index/AUROC indicates that a model can separate higher- from lower-risk patients; it does not mean that a predicted risk of, say 20%, corresponds to an observed event rate of 20%, which relates to accurate calibration. Preoperative counselling and consent rely on accurate absolute risks for shared decision making; calibration often deteriorates when models are applied outside their development setting [46]. This can therefore lead models to be misleading, and hence, models without sufficiently high calibration on external validation are of limited clinical value in the GO population. There may be an inherent ceiling to the level of discrimination that is possible in such models using routinely collected preoperative variables. A prospective multicentre UK study with 32 candidate variables (ineligible for this review due to the length of the outcome window) achieved an AUROC of 0.663 [47]. Taken together, this suggests that much of the variability may reflect unmeasured (and perhaps unmeasurable) factors.

Since our search was completed, a redeveloped logistic-regression version of the GO-SOAR calculator has been published with external validation in a prospective cohort of 416 patients from ten centres in six countries [48]. This study was published after completion of the systematic search, is not one of the 28 included studies and was not appraised with PROBAST; it is therefore presented as post-search contextual evidence only and does not form part of the systematic evidence base or the results reported above. This study showed good calibration (calibration slope 0.94) and moderate discrimination (AUROC 0.70). Its discrimination did not differ significantly from that of generic calculators such as ACS-NSQIP and POSSUM. Mortality estimates were based on only seven deaths in the validation cohort (24 in development), giving very wide confidence intervals (AUROC 0.75, 95% CI 0.57–0.94), and the morbidity outcome included all Clavien–Dindo grades rather than the severe complications most relevant to consent and most commonly modelled in the studies reviewed here. As validation was undertaken by the developing collaborative in predominantly European centres, this may not be valid in other settings.

These findings add to those from previous reviews of individual prognostic factors affecting GO surgery rather than multivariable models, which found increased age and advanced clinical stage most strongly associated with mortality, reflecting patient- and disease-specific factors [15]. The association between higher frailty scores and severe morbidity and mortality is consistent with previous reviews across multiple surgical specialities [49]. A previous systematic review of frailty in GO surgery concluded that preoperative frailty assessment is essential to predict adverse outcomes, reporting approximately four-fold higher odds of 30-day complications in frail patients [9]. Our conclusions are complementary but more cautious: an association with adverse outcomes does not in itself establish predictive performance, and in the present review, most frailty-based studies did not report discrimination, and none reported calibration or validation. Frailty assessment may therefore be a useful component of preoperative risk assessment, but its predictive performance within prediction models in the GO cohort is not yet established.

At present, no models can be recommended for standalone preoperative risk prediction in GO surgery. As there is limited calibration and external validation, the predicted probabilities given by such models should be interpreted cautiously and used to complement, not replace, clinical judgement. Frailty assessment is a reasonable adjunct to identify higher-risk patients. For ovarian cytoreductive surgery, the Cham nomogram is the most extensively tested, although it remains imperfect, and the prospectively developed international GO-SOAR model is methodologically notable but, within the included evidence, requires calibration assessment and external validation. Absolute risk estimates from the currently available models should be interpreted and communicated cautiously, particularly in populations or settings in which external calibration has not been demonstrated.

There is widespread variation in patient characteristics, disease burden and practice across and within countries [50,51,52,53]. The limited transportability observed to date suggests that a single model is unlikely to be directly applicable across all settings without local evaluation, and GO specialty-specific models would need to be independently externally validated in national or local settings, with full reporting of calibration and recalibration or model updating where required. This is required in order for models to be suitable for clinical counselling and decision-making, if indeed any model can achieve excellent predictive capability using measurable preoperative factors [46]. Standardised outcome definitions would help future comparison and quantitative synthesis, in particular, the threshold of complication severity (e.g., Clavien–Dindo ≥ III), the 30-day window, and whether mortality is modelled separately or as a composite.

## 5. Conclusions

Multiple risk-prediction models are used in gynaecological oncology; however, they demonstrate limited accuracy and validation within this cohort. It is not possible to clearly recommend any particular model in clinical practice, and further development and independent external validation of specialty-specific models in local settings are required to better inform preoperative counselling.

## Figures and Tables

**Table 1:** Characteristics of the included studies.

Author, Year	Country	Study Design	Sample Size	Data Source	Study Period
Alci 2025 [20]	Turkey	Retrospective cohort	179	Single centre	2015–2020
Barber 2015 [21]	USA	Retrospective cohort	751	NSQIP	2005–2013
Cham 2019 [22]	USA	Retrospective cohort	7029	NSQIP	2011–2015
Chambers 2021 [23]	USA	Retrospective cohort	141	Single centre	2014–2020
Delli Carpini 2024 [24]	Italy	Retrospective cohort	225	Multicentre	2018–2023
Gaba 2024 [18]	UK	Prospective cohort	1811	Multicentre	2021–2022
Giannini 2020 [25]	UK	Retrospective cohort	100	Single centre	2015–2017
Hermann 2022 [26]	USA	Retrospective cohort	21,215	NSQIP	2015–2017
Inci 2021 [27]	Germany	Prospective cohort	226	Single centre	2015–2017
Jiang 2022 [28]	China	Retrospective cohort	585	Single centre	2018–2020
Kohut 2018 [29]	USA	Retrospective cohort	12,831	NSQIP	2005–2013
Köse 2025 [30]	Turkey	Retrospective cohort	139	Single centre	2014–2023
Kumar 2016 [31]	USA	Retrospective cohort	620	Single centre	2003–2011
Latif 2018 [32]	USA	Retrospective cohort	2262	NSQIP	2006–2012
Levine 2023 [33]	USA	Retrospective cohort	886	NSQIP	2014–2020
Liu 2023 [34]	China	Prospective cohort	169	Single centre	2021–2022
Liu 2025 [35]	China	Retrospective cohort	922	Multicentre	2012–2023
Lomnytska 2021 [36]	Sweden	Prospective cohort	256	Single centre	2009–2018
Mah 2022 [37]	Canada	Retrospective cohort	259	Single centre	2016–2020
Nguyen 2025 [38]	Canada	Retrospective cohort	21,359	Multicentre	2009–2021
Nieuwenhuyzen-de Boer 2016 [39]	The Netherlands	Retrospective cohort	293	Multicentre	2004–2007
Park 2018 [40]	South Korea	Retrospective cohort	138	Single centre	2011–2017
Rivard 2016 [10]	USA	Retrospective cohort	1077	Single centre	2009–2013
Shim 2017 [19]	South Korea	Retrospective cohort	533	Single centre	2005–2015
Sideris 2025 [41]	UK	Retrospective cohort	161	Multicentre	2019–2022
Straubhar 2021 [42]	USA	Prospective cohort	700	Single centre	2015–2019
Szender 2015 [43]	USA	Retrospective cohort	577	Single centre	2012–2013
Vizzielli 2016 [44]	Italy	Retrospective cohort	555	Single centre	2006–2015

NSQIP, National Surgical Quality Improvement Program. USA, United States of America. UK, United Kingdom.

**Table 2:** Included population and surgical procedure.

Author, Year	Cancer Type	FIGO I–II	FIGO III–IV	Mean/Median Age (Years) ^a^	Surgical Approach	Procedure Type
Alci 2025 [20]	Ovarian	51	128	61	Open	PCS, ICS
Barber 2015 [21]	Ovarian	NR	NR	65+	Open	PCS
Cham 2019 [22]	Ovarian	NR	NR	61.2	Open	Oophorectomy ± hysterectomy, CRS
Chambers 2021 [23]	Ovarian, endometrial, other gynaecological	NR	NR	61.7	Open	PCS, ICS
Delli Carpini 2024 [24]	Vulvar	160	65	75	Open/perineal	Radical local excision, lymphadenectomy, reconstruction
Gaba 2024 [18]	Ovarian, endometrial, cervical, vulvar, vaginal	1101	710	61	Open, laparoscopic, robotic, vaginal	PCS
Giannini 2020 [25]	Endometrial	77	23	66.44	Laparoscopy, laparotomy	Total hysterectomy, oophorectomy ± lymph node dissection
Hermann 2022 [26]	Ovarian, endometrial, cervical, vulval, vaginal	NR	NR	59	Open, laparoscopic, vaginal	Hysterectomy
Inci 2021 [27]	Ovarian, endometrial, cervical, vulval, vaginal	NR	NR	59	NR	NR
Jiang 2022 [28]	Ovarian	NR	426	54.87	Open	PCS, ICS
Kohut 2018 [29]	Endometrial, cervical, ovarian, perineal	NR	NR	61	Open	PCS
Köse 2025 [30]	Ovarian	NR	139	60	Diagnostic laparoscopy, open	CRS
Kumar 2016 [31]	Ovarian	0	620	64.6	Open	PCS
Latif 2018 [32]	Ovarian	NR	NR	63.4	Open	PCS
Levine 2023 [33]	Vulval	NR	NR	57.9% aged ≥ 65	Vaginal	Radical vulvectomy ± inguinofemoral lymphadenectomy
Liu 2023 [34]	Ovarian, endometrial, cervical, fallopian	138	31	53	Open, laparoscopic	Radical hysterectomy, CRS, lymphadenectomy
Liu 2025 [35]	Ovarian	323	599	52.5	Laparoscopy, laparotomy	Upper abdominal surgery, enterectomy, lymphadenectomy
Lomnytska 2021 [36]	Ovarian	16	240	61.8	Laparoscopy, laparotomy	PCS, ICS
Mah 2022 [37]	Ovarian/fallopian/peritoneal, endometrial, cervical	77	130	75	Laparotomy	PCS, ICS, bowel resection, lymph node dissection
Nguyen 2025 [38]	Ovarian/peritoneal/tubal, endometrial, cervical, vulvar, vaginal	8332	7103	Median 61–72	Laparotomy	Total/radical hysterectomy, ovarian surgery, CRS
Nieuwenhuyzen-de Boer 2016 [39]	Ovarian	NI	293	64.7	Open	PCS
Park 2018 [40]	Cervical	NR	NR	43.1	Robotic-assisted	Radical hysterectomy/trachelectomy ± lymph node dissection, oophorectomy
Rivard 2016 [10]	NR	NR	NR	65+	Laparotomy	Hysterectomy with BSO, debulking, bowel resection
Shim 2017 [19]	Ovarian/tubal/peritoneal, endometrial, cervical, vulval, vaginal	NR	NR	DC 49; VC 52	Laparoscopy, laparotomy	CRS, hysterectomy, bowel resection, lymphadenectomy
Sideris 2025 [41]	Ovarian	42	119	66.5	Laparotomy	PCS, ICS, delayed CRS
Straubhar 2021 [42]	Ovarian	44	653	62	Open	PCS, ICS
Szender 2015 [43]	Ovarian, endometrial, cervical, vulval, vaginal	178	108	53	NR	NR
Vizzielli 2016 [44]	Ovarian	NI	555	59	Laparoscopy, laparotomy	PCS

^a^ Ages were approximated where studies reported grouped age categories. BSO, bilateral salpingo-oophorectomy; CRS, cytoreductive surgery; FIGO, International Federation of Gynaecology and Obstetrics; ICS, interval cytoreductive surgery; NI, not included; NR, not reported; PCS, primary cytoreductive surgery.

**Table 3:** Prediction-model characteristics.

Author, Year	Model	No. of Predictors ^a,b^	Predictors	Modelling Method	CD III+ Events (or Other)	Events per Predictor ^b^	Predicted Outcome
Multifactorial models							
Alci 2025 [20]	BayesNet algorithm	49	Age, comorbidities, surgical complexity, intraoperative interventions, length of stay, tumour-staging metrics, operative time, preoperative markers, NACT, ECOG	Machine learning (training/test sets)	40	0.8	CD III–V
Barber 2015 [21]	Preoperative personalised risk-assessment calculator	8	Preoperative markers, ascites, currently smoking, race	Multivariable logistic regression	123	15.3	CD III–V
Cham 2019 [22]	Ovarian cancer adverse perioperative outcome nomogram	7	SCS, age, elective surgery, preoperative ascites, bleeding disorder, albumin, ASA	Multivariable logistic regression	407	58.2	CD IV or 30-day mortality
Delli Carpini 2024 [24]	BMI + ACCI nomogram	2	BMI, ACCI	Binomial logistic regression	8	4.0	CD ≥ II (vs. none/grade I)
Gaba 2024 [18]	GO-SOAR surgical risk calculator	15	Age, ethnicity, BMI, haemoglobin, WCC, ASA, ECOG, primary cancer, FIGO, NACT, surgical history, intraoperative antibiotics, surgical modality, SCS	Machine learning; logistic regression	127	8.4	CD I–II; III–V; no morbidity
Jiang 2022 [28]	OC nomogram for CRS complications	4	Nomogram A: mFI, FIGO, BMI, SCS; Nomogram B: mFI, FIGO, NACT, SCS	Multivariable modelling	123 (Accordion IV)	30.7	Accordion grade IV
Kohut 2018 ^a^ [29]	Gynaecological oncology postoperative risk calculator	11, 17	Sepsis, functional status, SIRS, comorbidities, age, operative time, wound class, disseminated cancer, SCS, emergent case	Multivariable logistic regression	125, 784	11.4, 46.1	CD ≥ III
Kumar 2016 [31]	Nomogram for 30-day severe morbidity	6	Age, BMI, ASA, preoperative albumin, FIGO stage, surgical complexity	Multivariable modelling	138 (Accordion III/IV)	23.0	Accordion grade III or IV
Latif 2018 [32]	METRICS predictive model	6	Age, chronic hypertension requiring medication, ascites, WCC, haematocrit, serum creatinine	Multivariable logistic regression	194	32.3	CD ≥ III
Nieuwenhuyzen-de Boer 2016 [39]	30-day morbidity nomogram	3	Age, haemoglobin, WHO performance status	Univariable and multivariable Cox regression	113	37.6	CD II–IV
Park 2018 [40]	Modified surgical Apgar score	3	Estimated blood loss, lowest MAP, lowest HR	Linear-by-linear association	5	1.66	CD I–V
Rivard 2016 [10]	ACS-NSQIP surgical risk calculator	21	Sepsis, functional status, SIRS, comorbidities, age, operative time, wound class, disseminated cancer, SCS, emergent case	Multivariable logistic regression	219	10.4	CD II–V
Shim 2017 [19]	30-day morbidity nomogram	3	Age, operative time, albumin	Multivariable Cox regression	99	33.0	CD II–V
Sideris 2025 [41]	Augmented P-POSSUM (EFS + BMI)	3	Edmonton Frail Scale, BMI, P-POSSUM	Exploratory stepwise regression	16	5.3	CD I–V
Straubhar 2021 [42]	External validation of Cham nomogram	7	SCS, age, elective surgery, preoperative ascites, bleeding disorder, albumin, ASA	Logistic regression	51	7.28	CD IV
Szender 2015 [43]	ACS-NSQIP universal surgical risk calculator	14	Diagnosis, cancer stage, ECOG, SCS, estimated blood loss, NSQIP-tracked morbidities, disseminated cancer, prior abdominal surgery	Brier-score validation	84	6.0	CD I–II; III–V
Vizzielli 2016 [44]	Laparoscopic risk-adjusted model	4	ECOG, ascites, CA125, tumour load	Multivariable regression	102 (MSKCC grade ≥ 3)	25.5	MSKCC grade ≥ 3
Frailty models							
Chambers 2021 [23]	mFI	11	See Table S1	Univariable and multivariable logistic regression	18	1.64	CD ≥ II
Giannini 2020 [25]	mFI	11	See Table S1	Univariable and multivariable analysis	18	1.64	CD I–IV (overall); III–IV (severe)
Hermann 2022 [26]	mFI-5	5	See Table S1	Logistic regression	1864	372.8	CD III and IV
Inci 2021 [27]	Fried Frailty Score	5	Unintentional weight loss, exhaustion, grip strength, slow gait, low physical activity	Univariable and multivariable analysis	49	9.8	CD ≥ III
Levine 2023 [33]	mFI-5	5	See Table S1	Univariable and multivariable analysis	105	21	CD I–II; III–V
Liu 2023 [34]	Tilburg Frailty Indicator	15	Physical, psychological, social domains	Multivariable logistic regression	8	0.53	CD ≥ II
Mah 2022 [37]	mFI-5	5	See Table S1	Univariable and multivariable analysis	29	5.8	CD II–V; III–V
Nguyen 2025 ^a^ [38]	pFI; Johns Hopkins ACG	30; 32	Comorbidities, disability, sensory, cognitive, psychosocial and pharmaceutical domains, barriers to care, poverty	Multivariable logistic regression	1004	33.46, 31.375	CD II–IV
Biomarker and index-based models							
Köse 2025 [30]	Systemic Immune–Inflammation Index	6	Preoperative CA-125, SII, NLR, PLR, SIRI, MLR	Univariable and multivariable analysis	24	4.0	CD ≥ III
Liu 2025 [35]	Immunonutritional status nomogram	6	PNI > 46.73, FAR > 10.77, LMR > 3.70, hydrothorax, laparoscopy vs. laparotomy, enterectomy	Multivariable modelling	59	9.83	CD I–V
Lomnytska 2021 [36]	Peritoneal Cancer Index-based model	2	PCI and (SCS, cytoreduction score or albumin)	Univariable and multivariable analysis	62	31	CD ≥ III

^a^ Where two values are given (Kohut 2018 [29]; Nguyen 2025 [38]), these correspond to two separate models reported in the same study. ^b^ Number of predictors (or index components) in each model as reported by the study; for most models, this reflects retained predictors, as candidate predictors and predictor parameters were inconsistently reported. Events per predictor (EPP) = outcome events divided by this number; values are therefore approximate and may overstate events per candidate predictor parameter. The Accordion and MSKCC systems are recognised derivatives of the Clavien–Dindo classification. ACCI, Age-Adjusted Charlson Comorbidity Index; ACG, Adjusted Clinical Groups; ACS-NSQIP, American College of Surgeons National Surgical Quality Improvement Program; ASA, American Society of Anaesthesiologists grade; BMI, body mass index; CA-125, cancer antigen 125; CD, Clavien–Dindo grade; CRS, cytoreductive surgery; ECOG, Eastern Cooperative Oncology Group performance status; EFS, Edmonton Frail Scale; FAR, fibrinogen-to-albumin ratio; FIGO, International Federation of Gynaecology and Obstetrics; GO-SOAR, Gynaecological Oncology Surgical Outcomes and Audit Research collaborative (model listed as PROMEGO); HR, heart rate; LMR, lymphocyte-to-monocyte ratio; MAP, mean arterial pressure; METRICS, Mortality and sErious morbidity after ovarian Cancer Surgery; mFI, modified frailty index; MLR, monocyte-to-lymphocyte ratio; MSKCC, Memorial Sloan Kettering Cancer Center surgical secondary-events grade; NACT, neoadjuvant chemotherapy; NLR, neutrophil-to-lymphocyte ratio; OC, ovarian cancer; PCI, Peritoneal Cancer Index; pFI, physiological frailty index; PLR, platelet-to-lymphocyte ratio; PNI, prognostic nutritional index; P-POSSUM, Portsmouth Physiological and Operative Severity Score for the enUmeration of Mortality and Morbidity; SCS, surgical complexity score; SII, Systemic Immune–Inflammation Index; SIRI, systemic inflammatory response index; SIRS, systemic inflammatory response syndrome; WCC, white cell count; WHO, World Health Organization.

**Table 4:** Model performance.

Author, Year	Model	Discrimination Metric	AUROC/c-Statistic	Internal Validation	Calibration
Multifactorial models					
Alci 2025 [20]	BayesNet algorithm	AUROC (c-statistic)	0.863	Random split sample	Not reported
Barber 2015 [21]	Preoperative personalised risk-assessment calculator	AUROC (c-statistic)	0.725	Not reported	Hosmer–Lemeshow *p* = 0.53
Cham 2019 [22]	Ovarian cancer adverse perioperative outcome nomogram	AUROC (c-statistic)	0.71	Bootstrap (200 resamples); 10-fold cross-validation ×20	Calibration plot (predicted vs. observed)
Delli Carpini 2024 [24]	BMI + ACCI nomogram	Not reported	Not reported	Not reported	Not reported
Gaba 2024 ^a^ [18]	GO-SOAR surgical risk calculator	AUROC (c-statistic)	Morbidity (neural network) 0.94; mortality (logistic regression) 0.66	Leave-one-out CV; Monte Carlo CV (1000 iterations)	Not reported
Jiang 2022 [28]	OC nomogram for CRS complications	AUROC (c-statistic)	0.75 overall; 0.79 severe	Bootstrapping	Hosmer–Lemeshow (*p* not reported); calibration curve
Kohut 2018 ^b^ [29]	Gynaecological oncology postoperative risk calculator	AUROC (c-statistic)	Mortality 0.86; major morbidity 0.68	Not reported	Not reported
Kumar 2016 [31]	Nomogram for 30-day severe morbidity	AUROC (c-statistic)	0.648	Bootstrap (1000 resamples)	Calibration plot (predicted vs. observed)
Latif 2018 [32]	METRICS predictive model	AUROC (c-statistic)	0.66	Ten-fold cross-validation	Not reported
Nieuwenhuyzen-de Boer 2016 [39]	30-day morbidity nomogram	AUROC (c-statistic)	0.62	Bootstrapping (200); shrinkage factor 0.78	Not reported
Park 2018 [40]	Modified surgical Apgar score	Not reported	Not reported	Not reported	Not reported
Rivard 2016 [10]	ACS-NSQIP surgical risk calculator	AUROC (c-statistic)	Overall 0.635; serious complication 0.644; death 0.851	Not reported	Not reported
Shim 2017 [19]	30-day morbidity nomogram	AUROC (c-statistic)	DC 0.656; VC 0.674	Bootstrap (1000); split sample	Hosmer–Lemeshow *p* = 0.774 (DC), 0.614 (VC)
Sideris 2025 [41]	Augmented P-POSSUM (EFS + BMI)	AUROC (c-statistic)	P-POSSUM 0.529; augmented model 0.655	Bootstrapping (2000 resamples)	Calibration plot (predicted vs. observed)
Straubhar 2021 [42]	External validation of Cham nomogram	AUROC (c-statistic)	0.715	Bootstrap (1000 resamples)	Calibration curve (predicted vs. observed)
Szender 2015 [43]	ACS-NSQIP universal surgical risk calculator	AUROC (c-statistic)	0.5	Not reported	Not reported
Vizzielli 2016 [44]	Laparoscopic risk-adjusted model	AUROC (c-statistic)	0.79	Bootstrap (200); shrinkage factor 0.94	Hosmer–Lemeshow *p* = 0.729
Frailty models					
Chambers 2021 [23]	mFI	Not reported	Not reported	Not reported	Not reported
Giannini 2020 [25]	mFI	Not reported	Not reported	Not reported	Not reported
Hermann 2022 [26]	mFI-5	AUROC (c-statistic)	Mortality 0.801; severe complication 0.732	Not reported	Not reported
Inci 2021 [27]	Fried Frailty Score	AUROC (c-statistic)	Not reported	Not reported	Not reported
Levine 2023 [33]	mFI-5	Not reported	Not reported	Not reported	Not reported
Liu 2023 [34]	Tilburg Frailty Indicator	AUROC (c-statistic)	Not reported	Not reported	Not reported
Mah 2022 [37]	mFI-5, age, BMI	AUROC (c-statistic)	0.92	Not reported	Not reported
Nguyen 2025 ^b^ [38]	pFI; Johns Hopkins ACG	AUROC (c-statistic)	pFI 0.71; ACG 0.70	Not reported	Not reported
Biomarker and index-based models					
Köse 2025 [30]	CA125, Systemic Immune–Inflammation Index	AUROC (c-statistic)	CA125 0.766; SII 0.74; NLR 0.719; PLR 0.668; SIRI 0.651	Bootstrap (1000 resamples)	Not reported
Liu 2025 [35]	Immunonutritional status nomogram	AUROC (c-statistic)	Training 0.723; validation 0.709	Training/validation split (7:3)	Hosmer–Lemeshow *p* = 0.987 (training), 0.310 (validation)
Lomnytska 2021 [36]	Peritoneal Cancer Index-based model	AUROC (c-statistic)	Not reported ^c^	Not reported	Not reported

^a^ The neural-network AUROC of 0.94 is a multiclass value and is not comparable with the binary c-statistics reported for other models. ^b^ Where two values are given (Kohut 2018 [29]; Nguyen 2025 [38]), these correspond to two separate models reported in the same study. ^c^ Discrimination was assessed graphically only. ACG, Adjusted Clinical Groups; ACS-NSQIP, American College of Surgeons National Surgical Quality Improvement Program; AUROC, area under the receiver operating characteristic curve; ACCI, Age adjusted Charlson Comorbidity Index; BMI, body mass index; CA125, cancer antigen 125; CV, cross-validation; CRS, cytoreductive surgery; DC, development cohort; GO-SOAR, Gynaecological Oncology Surgical Outcomes and Audit Research collaborative; METRICS, Mortality and sErious morbidity after ovarian Cancer Surgery; mFI, modified frailty index; NLR, neutrophil-to-lymphocyte ratio; OC, ovarian cancer; pFI, physiological frailty index; PLR, platelet-to-lymphocyte ratio; P-POSSUM, Portsmouth Physiological and Operative Severity Score for the enUmeration of Mortality and Morbidity; SII, Systemic Immune–Inflammation index; SIRI, systemic inflammatory response index; VC, validation cohort.

## Data Availability

No new data were created in this study. All data extracted from the included studies are presented within the article and Supplementary Materials. The review protocol is registered with PROSPERO (CRD420251179495) and is publicly available at https://www.crd.york.ac.uk/prospero/ (accessed on 3 October 2026).

## Supplementary Materials

The following supporting information can be downloaded at https://www.mdpi.com/article/10.3390/jcm15207948/s1: Supplementary S.1, PRISMA Checklist; Supplementary S.2, search strategy; Table S1, variables comprising the modified frailty index; Table S2, outcomes reported; Table S3, PROBAST risk of bias and applicability.

## Abbreviations

The following abbreviations are used in this manuscript:

ACG	Adjusted Clinical Groups
ACS-NSQIP	American College of Surgeons National Surgical Quality Improvement Program
AUROC	Area under the receiver operating characteristic curve
BMI	Body mass index
CI	Confidence interval
CRS	Cytoreductive surgery
FIGO	International Federation of Gynaecology and Obstetrics
GO	Gynaecological oncology
GO-SOAR	Gynaecological Oncology Surgical Outcomes and Audit Research collaborative
mFI	Modified frailty index
MSKCC	Memorial Sloan Kettering Cancer Center
PCI	Peritoneal Cancer Index
PICO	Population, intervention, comparison, outcomes
POSSUM	Physiological and Operative Severity Score for the enUmeration of Mortality and Morbidity
P-POSSUM	Portsmouth POSSUM
PRISMA	Preferred Reporting Items for Systematic Reviews and Meta-Analyses
PROBAST	Prediction model Risk of Bias Assessment Tool
SII	Systemic Immune–Inflammation Index
TRIPOD	Transparent Reporting of a multivariable prediction model for Individual Prognosis or Diagnosis
