# Supplementary material for: Predictive Models of Morbidity and Mortality in Gynaecological Cancer Surgery: A Systematic Review
Source: J Clin Med. 2026 Oct 14;15(20):7948. doi: 10.3390/jcm15207948 (PMC13706611; doi:10.3390/jcm15207948)
Supplement: Supplementary file 1 [file jcm-15-07948-s001.zip › jcm-4571211-supplementary.pdf]

Journal of Clinical Medicine

MDPI

Systematic Review

S1

S1.1. PRISMA Checklist

Section and Topic	Item #	Checklist item	Location where item is reported
**TITLE**			
Title	1	Identify the report as a systematic review.	Page 1
**ABSTRACT**			
Abstract	2	See the PRISMA 2020 for Abstracts checklist.	Page 1
**INTRODUCTION**			
Rationale	3	Describe the rationale for the review in the context of existing knowledge.	Page 2
Objectives	4	Provide an explicit statement of the objective(s) or question(s) the review addresses.	Page 2
**METHODS**			
Eligibility criteria	5	Specify the inclusion and exclusion criteria for the review and how studies were grouped for the syntheses.	Page 2-3
Information sources	6	Specify all databases, registers, websites, organisations, reference lists and other sources searched or consulted to identify studies. Specify the date when each source was last searched or consulted.	Page 3
Search strategy	7	Present the full search strategies for all databases, registers and websites, including any filters and limits used.	Appendix A Page 16
Selection process	8	Specify the methods used to decide whether a study met the inclusion criteria of the review, including how many reviewers screened each record and each report retrieved, whether they worked independently, and if applicable, details of automation tools used in the process.	Page 3-4

Section and Topic	Item #	Checklist item	Location where item is reported
Data collection process	9	Specify the methods used to collect data from reports, including how many reviewers collected data from each report, whether they worked independently, any processes for obtaining or confirming data from study investigators, and if applicable, details of automation tools used in the process.	Page 3-4
Data items	10a	List and define all outcomes for which data were sought. Specify whether all results that were compatible with each outcome domain in each study were sought (e.g. for all measures, time points, analyses), and if not, the methods used to decide which results to collect.	Page 3-4
	10b	List and define all other variables for which data were sought (e.g. participant and intervention characteristics, funding sources). Describe any assumptions made about any missing or unclear information.	Page 3-4
Study risk of bias assessment	11	Specify the methods used to assess risk of bias in the included studies, including details of the tool(s) used, how many reviewers assessed each study and whether they worked independently, and if applicable, details of automation tools used in the process.	Page 4
Effect measures	12	Specify for each outcome the effect measure(s) (e.g. risk ratio, mean difference) used in the synthesis or presentation of results.	Page 4<br>Tables 3–4<br>Pages 8-11
Synthesis methods	13a	Describe the processes used to decide which studies were eligible for each synthesis (e.g. tabulating the study intervention characteristics and comparing against the planned groups for each synthesis (item #5)).	Page 3<br>Tables 3–4<br>Pages 8-11
	13b	Describe any methods required to prepare the data for presentation or synthesis, such as handling of missing summary statistics, or data conversions.	Page 3-4
	13c	Describe any methods used to tabulate or visually display results of individual studies and syntheses.	Tables 1–4, Pages 5-7 and Pages 8-11<br>Table A3<br>Page 18

Section and Topic	Item #	Checklist item	Location where item is reported
	13d	Describe any methods used to synthesize results and provide a rationale for the choice(s). If meta-analysis was performed, describe the model(s), method(s) to identify the presence and extent of statistical heterogeneity, and software package(s) used.	Page 3-4<br>Tables 3–4, Pages 8-11<br>Table A3<br>Page 18
	13e	Describe any methods used to explore possible causes of heterogeneity among study results (e.g. subgroup analysis, meta-regression).	Pages 3-4<br>Tables 3–4, Pages 8-11
	13f	Describe any sensitivity analyses conducted to assess robustness of the synthesized results.	Page 4<br>Appendix Table A3<br>Page 18
Reporting bias assessment	14	Describe any methods used to assess risk of bias due to missing results in a synthesis (arising from reporting biases).	Appendix Table A3<br>Page 18
Certainty assessment	15	Describe any methods used to assess certainty (or confidence) in the body of evidence for an outcome.	Not Applicable
**RESULTS**			
Study selection	16a	Describe the results of the search and selection process, from the number of records identified in the search to the number of studies included in the review, ideally using a flow diagram.	Page 5<br>Figure 2 (PRISMA flow diagram).

Section and Topic	Item #	Checklist item	Location where item is reported
	16b	Cite studies that might appear to meet the inclusion criteria, but which were excluded, and explain why they were excluded.	Page 3
Study characteristics	17	Cite each included study and present its characteristics.	Pages 4-7
Risk of bias in studies	18	Present assessments of risk of bias for each included study.	Page 13 Appendix Table A3 Page 18
Results of individual studies	19	For all outcomes, present, for each study: (a) summary statistics for each group (where appropriate) and (b) an effect estimate and its precision (e.g. confidence/credible interval), ideally using structured tables or plots.	Page 6-13 Tables 2–4, Pages 6-11
Results of syntheses	20a	For each synthesis, briefly summarise the characteristics and risk of bias among contributing studies.	Pages 10-13
	20b	Present results of all statistical syntheses conducted. If meta-analysis was done, present for each the summary estimate and its precision (e.g. confidence/credible interval) and measures of statistical heterogeneity. If comparing groups, describe the direction of the effect.	Pages 8-11 Tables 3-4 Appendix Table A3 Table A2
	20c	Present results of all investigations of possible causes of heterogeneity among study results.	Not Applicable
	20d	Present results of all sensitivity analyses conducted to assess the robustness of the synthesized results.	Not Applicable
Reporting biases	21	Present assessments of risk of bias due to missing results (arising from reporting biases) for each synthesis assessed.	Not

Section and Topic	Item #	Checklist item	Location where item is reported
			Applicable
Certainty of evidence	22	Present assessments of certainty (or confidence) in the body of evidence for each outcome assessed.	Page 13<br>Appendix Table A3<br>Page 18
**DISCUSSION**			
Discussion	23a	Provide a general interpretation of the results in the context of other evidence.	Pages 13
	23b	Discuss any limitations of the evidence included in the review.	Page 13
	23c	Discuss any limitations of the review processes used.	Page 13
	23d	Discuss implications of the results for practice, policy, and future research.	Page 13-14
**OTHER INFORMATION**			
Registration and protocol	24a	Provide registration information for the review, including register name and registration number, or state that the review was not registered.	Page 2
	24b	Indicate where the review protocol can be accessed, or state that a protocol was not prepared.	Page 2
	24c	Describe and explain any amendments to information provided at registration or in the protocol.	Page 2
Support	25	Describe sources of financial or non-financial support for the review, and the role of the funders or sponsors in the review.	Page 15
Competing interests	26	Declare any competing interests of review authors.	Page 15
Availability of data, code and other materials	27	Report which of the following are publicly available and where they can be found: template data collection forms; data extracted from included studies; data used for all analyses; analytic code; any other materials used in the review.	Page 15

From: Page MJ, McKenzie JE, Bossuyt PM, Boutron I, Hoffmann TC, Mulrow CD, et al. The PRISMA 2020 statement: an updated guideline for reporting systematic reviews. BMJ 2021;372:n71. doi: 10.1136/bmj.n71. This work is licensed under CC BY 4.0. To view a copy of this license, visit https://creativecommons.org/licenses/by/4.0/

S1.2. Search Strategy

PubMed

Search: Predict* AND (Mortality OR Morbidity) AND (postoperative OR perioperative) AND (gyne* OR gyna* OR cytoredu*) AND cancer AND risk. Filters: English, Humans.

"predict*"[All Fields]

AND ("mortality"[MeSH Terms] OR "mortality"[All Fields] OR "mortalities"[All Fields] OR "mortality"[MeSH Subheading] OR ("epidemiology"[MeSH Subheading] OR "epidemiology"[All Fields] OR "morbidity"[All Fields] OR "morbidity"[MeSH Terms] OR "morbid"[All Fields] OR "morbidities"[All Fields] OR "morbids"[All Fields]))

AND ("postoperative period"[MeSH Terms] OR ("postoperative"[All Fields] AND "period"[All Fields]) OR "postoperative period"[All Fields] OR "postop"[All Fields] OR "postoperative"[All Fields] OR "postoperatively"[All Fields] OR "postoperatives"[All Fields] OR ("perioperative"[All Fields] OR "perioperatively"[All Fields]))

AND ("gyne*"[All Fields] OR "gyna*"[All Fields] OR "cytoredu*"[All Fields])

AND ("cancer s"[All Fields] OR "cancerated"[All Fields] OR "canceration"[All Fields] OR "cancerization"[All Fields] OR "cancerized"[All Fields] OR "cancerous"[All Fields] OR "neoplasms"[MeSH Terms] OR "neoplasms"[All Fields] OR "cancer"[All Fields] OR "cancers"[All Fields])

AND ("risk"[MeSH Terms] OR "risk"[All Fields])

AND ((humans[Filter]) AND (english[Filter]))

MEDLINE

(exp predict/ or predict*.af.)

AND (exp mortality/ or mortality.af. or exp morbidity/ or morbidity.af.)

AND ((gyne* or gyna* or cytoredu*).af.)

AND (exp cancer/ or cancer.af.)

AND (exp risk/ or risk.af.)

AND (exp Perioperative Period/ or Perioperative.af. or exp Postoperative Complications/ or postoperative.af. or post-operative.af.)

Filters: [English] [Humans]

EMBASE

'predict'/exp OR predict*

AND 'mortality'/exp OR mortality OR 'morbidity'/exp OR morbidity

AND gyne* OR gyna* OR cytoredu*

AND 'cancer'/exp OR cancer

AND 'risk'/exp OR risk

AND 'perioperative period'/exp OR perioperative OR 'postoperative complications'/exp OR postoperative OR 'post operative' OR 'outcome assessment'

AND [Humans]/lim AND [English]/lim

Databases were searched on 20 November 2025 with no date restriction.

Table S1. Variables comprising the modified frailty index.

mFI-5 Variables	mFI-11 Variables
Non-independent functional status	Non-independent functional status
History of diabetes mellitus	History of diabetes mellitus
History of chronic obstructive pulmonary disease	History of chronic obstructive pulmonary disease
History of congestive heart failure	History of congestive heart failure
Hypertension requiring the use of medication	Hypertension requiring the use of medication
	History of percutaneous coronary intervention, cardiac surgery or angina
	History of myocardial infarction
	Peripheral vascular disease or rest pain
	Impaired sensorium
	Transient ischaemic attack or cerebrovascular accident without residual deficit
	Cerebrovascular accident with deficit

mFI, modified frailty index.

Table S2. Outcomes reported by the included studies.

Author, Year	Morbidity	30-Day Mortality	Other Reported Outcomes
Alci 2025	CD III–V	–	–
Barber 2015	CD III–V	–	–
Cham 2019	CD IV or 30-day mortality	Yes	–
Chambers 2021	CD ≥II	Yes	ICU admission, time to discharge
Delli Carpini 2024	CD ≥II	–	–
Gaba 2024	CD I–II; III–V	Yes	–
Giannini 2020	CD I–IV; III–IV	–	–
Hermann 2022	CD III and IV	Yes	–

Author, Year	Morbidity	30-Day Mortality	Other Reported Outcomes
Inci 2021	CD ≥IIIb	Yes	–
Jiang 2022	Accordion IV	–	90-day mortality
Kohut 2018 [a]	CD ≥III	Yes	–
Köse 2025	CD ≥III	Yes	–
Kumar 2016	Accordion III or IV	Yes	90-day mortality
Latif 2018	CD ≥III	Yes	–
Levine 2023	CD I–II; III–V	–	Minor complications
Liu 2023	CD ≥II	Yes	3-day Quality of Recovery (QoR-15)
Liu 2025	CD I–V	–	–
Lomnytska 2021	CD ≥IIIb	Yes	Reoperation
Mah 2022	CD II–V; III–V	–	–
Nguyen 2025	CD II–IV	Yes	90-day mortality
Nieuwenhuyzen-de Boer 2016	CD II–IV	Yes	–
Park 2018	CD I–V	Yes	–
Rivard 2016	CD II–V	Yes	–
Shim 2017	CD II–V	Yes	–
Sideris 2025	CD I–V	Yes	–
Straubhar 2021	CD IV	Yes	–
Szender 2015	CD I–II; III–V	Yes	–
Vizzielli 2016	MSKCC grade ≥3	Yes	–

–, not reported. [a] This study reports two separate models for mortality and major morbidity, respectively. CD, Clavien–Dindo grade; ICU, intensive care unit; MSKCC, Memorial Sloan Kettering Cancer Center surgical secondary events grading system.

Table S3. PROBAST risk of bias and applicability assessment.

Study	Participant Selection	Predictors	Outcome	Analysis	Overall Risk of Bias	Applicability Concerns
Alci 2025	L	H	L	H	H	L
Barber 2015	M	L	L	H	H	L
Cham 2019	L	L	L	L	L	L
Chambers 2021	L	L	L	H	H	L
Delli Carpini 2024	L	L	L	H	H	L

Study	Participant Selection	Predictors	Outcome	Analysis	Overall Risk of Bias	Applicability Concerns
Gaba 2024	L	L	L	H	H	L
Giannini 2020	L	L	L	H	H	L
Hermann 2022	L	L	L	H	H	L
Inci 2021	L	L	L	H	H	L
Jiang 2022	L	L	L	L	L	L
Kohut 2018	L	L	L	H	H	L
Köse 2025	L	L	L	H	H	L
Kumar 2016	L	L	L	L	L	L
Latif 2018	L	L	L	H	H	L
Levine 2023	L	L	L	H	H	L
Liu 2023	M	L	L	H	H	L
Liu 2025	L	L	L	L	L	L
Lomnytska 2021	L	L	L	H	H	L
Mah 2022	M	L	L	H	H	L
Nguyen 2025	L	H	L	H	H	L
Nieuwenhuyzen-de Boer 2016	L	L	L	H	H	L
Park 2018	L	M	L	H	H	L
Rivard 2016	L	M	L	H	H	L
Shim 2017	L	L	L	L	L	L
Sideris 2025	L	L	L	L	L	L
Straubhar 2021	L	L	L	L	L	L
Szender 2015	L	L	L	H	H	L
Vizzielli 2016	L	L	L	L	L	L

L, low; M, moderate; H, high. Overall risk of bias and applicability concerns follow standard PROBAST signalling. PROBAST was applied at the study (publication) level; where a study reported more than one model or analysis (e.g. separate models, or development and validation analyses), a single judgement is presented for each domain across the reported analyses.
